# RETRACTED: Saleh et al. Cell Cycle Arrest in Different Cancer Cell Lines (Liver, Breast, and Colon) Induces Apoptosis Under the Influence of the Chemical Content of *Aeluropus lagopoides* Leaf Extracts. *Molecules* 2019, *24*, 507

**DOI:** 10.3390/molecules31162805

**Published:** 2026-08-12

**Authors:** Kamel A. Saleh, Tahani H. Albinhassan, Serage Eldin I. Elbehairi, Mohammed A. Alshehry, Mohammad Y. Alfaifi, Adel M. Al-Ghazzawi, Mohamed A. Al-Kahtani, Abdullah D. A. Alasmari

**Affiliations:** 1 Department of Biology, Science College, King Khalid University, Abha P.O. Box 9004, Saudi Arabia; dr_ksaleh@yahoo.com (K.A.S.); seragalone@gmail.com (S.E.I.E.); alshehri44@gmail.com (M.A.A.); almashnawe@hotmail.com (M.Y.A.); mueaalqahtani@kku.edu.sa (M.A.A.-K.); 2 Department of Chemistry, Science College, King Khalid University, Abha P.O. Box 9004, Saudi Arabia; algawazy@kku.edu.sa; 3 Asser Toxicology Center, King Abduallah Street, 61441, Abha P.O. Box 1988, Saudi Arabia; azalasmari@gmail.com

The journal retracts the article “Cell Cycle Arrest in Different Cancer Cell Lines (Liver, Breast, and Colon) Induces Apoptosis under the Influence of the Chemical Content of Aeluropus lagopoides Leaf Extracts” [1], cited above.

Following publication, concerns were brought to the attention of the Editorial Office regarding the integrity of an image presented in this publication [1].

In accordance with the journal standard procedures the Editorial Office and Editorial Board conducted an investigation that identified image duplication within Figure 2, and concerns regarding the accuracy of the authorship list. Although the authors cooperated throughout the process, the concerns identified could not be satisfactorily resolved based on the information and materials provided.

Consequently, the Editorial Board has lost confidence in the reliability of the findings and has decided to retract this publication, as per MDPI’s retraction policy.

This retraction was approved by the Editor-in-Chief of the journal *Molecules*. 

The authors have been informed of this retraction but did not provide a comment on this decision.
